# Supplementary material for: Evolutionary analysis across mammals reveals distinct classes of long non-coding RNAs
Source: Genome Biol. 2016 Feb 2;17:19. doi: 10.1186/s13059-016-0880-9 (PMC4739325; doi:10.1186/s13059-016-0880-9)
Supplement: Additional File 1: — Supplementary figures and tables. (PDF 3.85 MB) [file 13059_2016_880_MOESM1_ESM.pdf]

Supplementary Table 1

Supplementary Table 1. RNA-Sequencing libraries used in study

**Species**	**Strain**	**Assembly**	**Cell type**	**Number of fragments sequenced**	**Number of aligned fragments** (duplicates removed)	**SRA accession**
Mouse	*120SvEv*	mm9	naïve ESC	180,535,866	118,386,301	
Mouse	*120SvEv*	mm9	primed epiSC	180,368,378	110,377,225	
Mouse	*NOD*	mm9	naïve ESC	141,615,128	94,816,294	
Mouse	*NOD*	mm9	primed epiSC	177,918,230	102,394,440	
Mouse	*cast*	mm9	naïve ESC	199,168,080	158,066,464	
Mouse	*cast*	mm9	primed epiSC	224,000,150	157,372,110	
Rat		rn5	naïve iPS	247,087,648	100,883,472	
Rat		rn5	primed iPS	114,987,318	80,516,323	
Chimpanzee		pantro4	iPS	159,906,000	108,736,080	SRR873623, SRR873624, SRR873625, SRR873626
Bonobo		pantro4	iPS	239,033,834	162,543,008	SRR873626, SRR873629, SRR873628, SRR873627
Human		hg19	iPS	244,014,732	201,066,988	

Table S1. **RNA-Sequencing libraries used in study**. The table shows number of fragments sequenced and aligned to assembly after optical duplicates were removed. Rows highlighted in gray indicate downloaded data. All other data was generated for this study.

Supplementary Figure 1

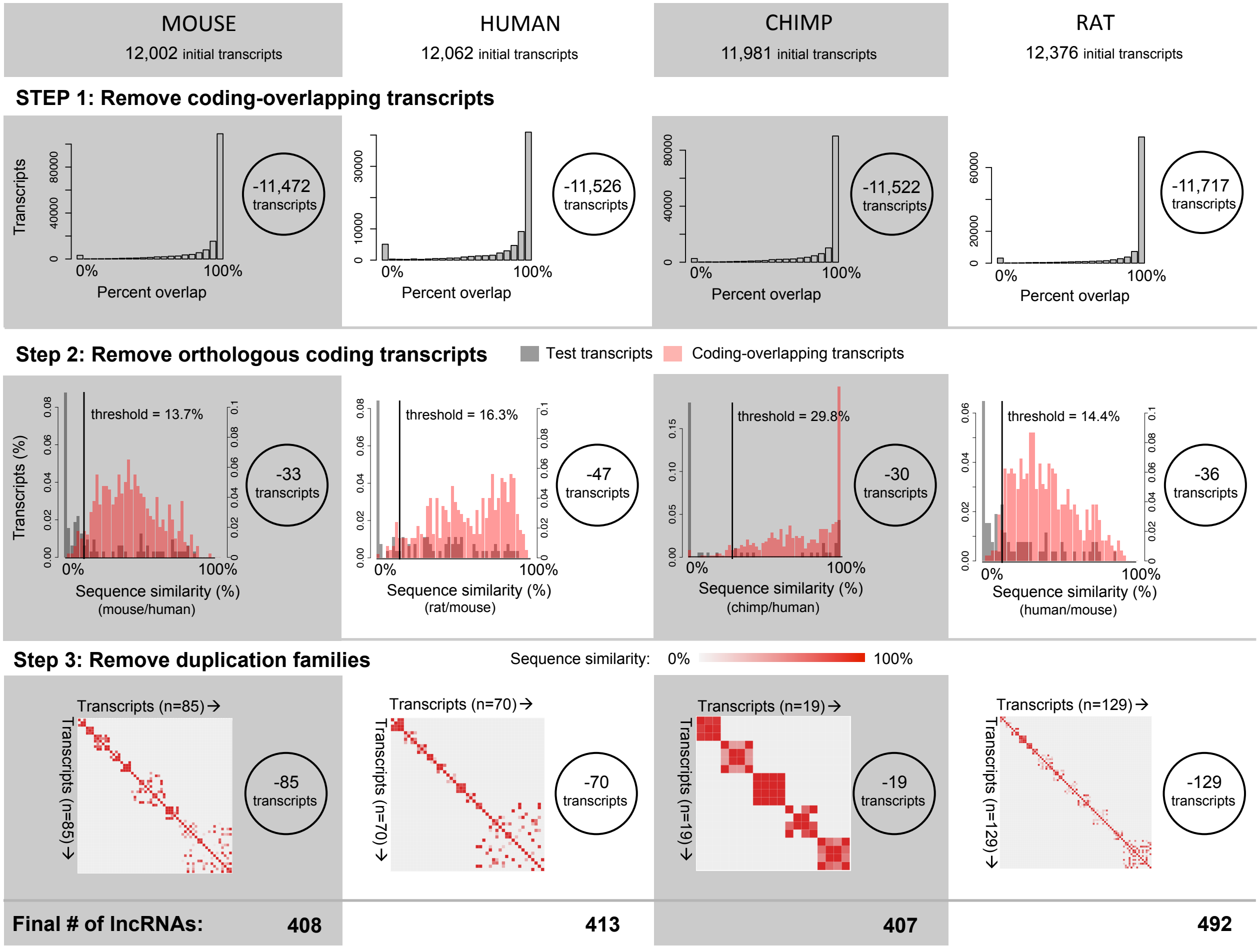

Figure S1. **slncky filters high quality set of lncRNAs from mouse, rat, chimp, and human RNA-Seq data**. Top row: Histogram of percent exonic overlap of reconstructed transcripts with annotated coding genes. Number of transcripts removed are shown inside circles (right). Middle row: Histogram of exonic sequence similarity between coding-overlapping transcripts that align to syntenic coding genes (red) and reconstructed transcripts that align to a syntenic coding gene (gray). Distribution of sequence similarity for coding-overlapping transcripts is used as a positive distribution to define empirical 5% threshold used for filtering. Bottom row: Heatmap of sequence similarity between reconstructed transcripts that align significantly to each other. Only significant alignments are displayed.

Supplementary Figure 2

A

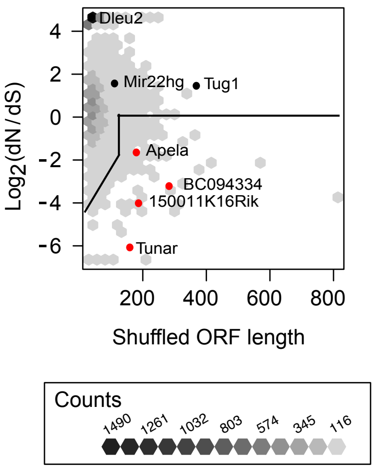

B

Gene	Longest ORF (bp)	dN	dS	dN/dS	RNACode *P*-values
Tunar	147	0.003	0.22	**.014**	**1.19e-14**
150011K16Rik	171	0.01	0.14	**.059**	**2.78e-04**
BC094334	255	0.02	0.15	**.103**	**5.90e-10**
Apela	165	0.05	0.15	**.308**	**2.00e-03**

C

Gene	Longest ORF (bp)	dN	dS	dN/dS	RNACode *P*-values
Tug1	330	0.04	0.02	2.693	**4.34e-06**
Malat1	NA	-	-	-	**2.28e-04**
Cyrano	NA	-	-	-	**1.10e-03**
Mir22hg	105	0.10	0.04	2.905	**6.00e-03**
Dleu2	33	0.16	0.00	inf	**6.003-03**

Figure S2. slncky flags novel, conserved open reading frames (ORFs) while maintaining sensitivity for identifying conserved lncRNAs. A) Binned scatterplot of lengths (x-axis) and log2(dN / dS ratios) (y-axis) across ORFs found in alignments of shuffled transcripts. This distribution was used as a null distribution for determining empirical P-values of conserved ORFs found in true lncRNA orthologs (Methods). Thick line shows cutoff for P = 0.05 as a function of ORF length. For long ORFs, for which less than 100 length-matched random ORFs existed, we could not accurately estimate the P-value cutoff, so we set the log2(dN / dS ratio) cutoff to 1. Labeled black points are true lncRNAs flagged as coding by RNACode; labeled red points are conserved ORFs flagged by slncky. B) Table of orthologous “lncRNAs” containing conserved ORFs with significant dN / dS ratios (bold). These four ORFs also have significant RNACode P-values (bold). C) Table of known lncRNAs with significant coding potential by RNACode (bold) but insignificant dN / dS ratios.

Supplementary Figure 3

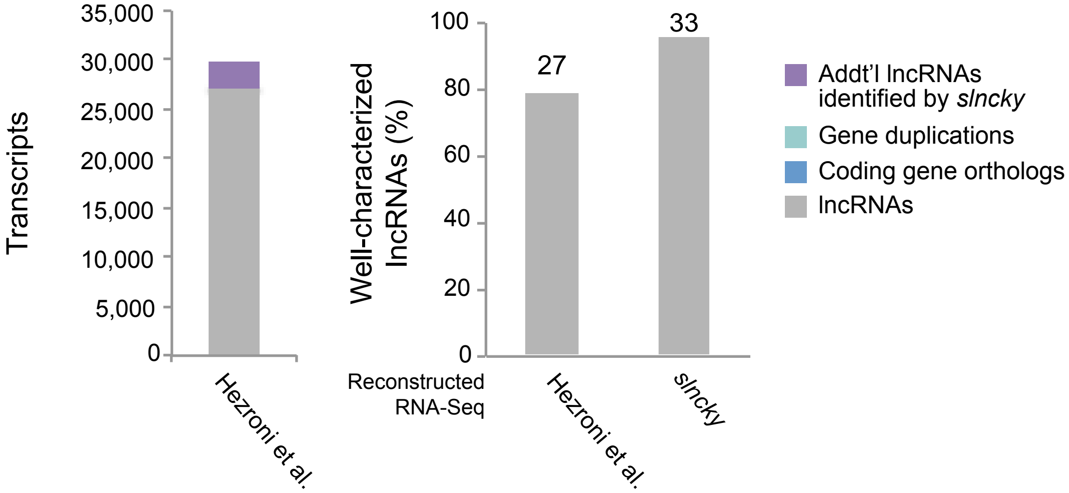

Figure S3. **slncky performs comparably to PLAR but recovers more well-characterized lncRNAs.** Left: Comparison of PLAR-filtered lncRNAs to slncky results. Number of transcripts also annotated as a lncRNA by slncky (gray), number removed by slncky as gene duplication or coding (light and dark blue), and number of additional transcripts annotated as a lncRNA by slncky but not the previous pipeline (purple). Right: Percentage of well-characterized lncRNAs identified by PLAR compared to slncky results. Numbers above bars denote absolute number of lncRNAs.

Supplementary Figure 4

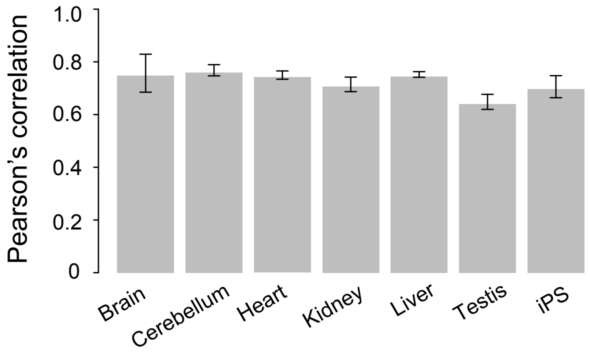

Figure S4. **iPS cells are comparable across mammals.** Barplot of Pearson's correlation of log10(FPKM) values (for all genes where FPKM > 0) between every pair of mouse and human samples across somatic tissue (Merkin et al.) and within our iPS data.

Supplementary Figure 5

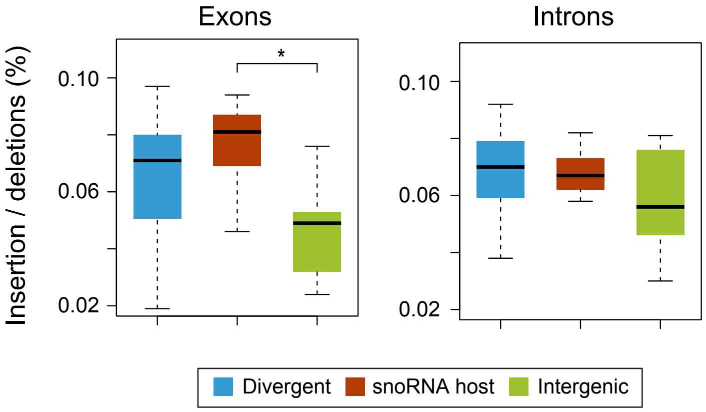

Figure S5. snoRNA host genes have excess of exonic, but not intronic indels, compared to intergenic lncRNAs. Boxplots of percentages of indels rate across exons (left) and introns (right) of divergent (blue), snoRNA host (purple), and intergenic (green) lncRNAs. * denotes $P < 0.05$ (t-test).

Supplementary Figure 6

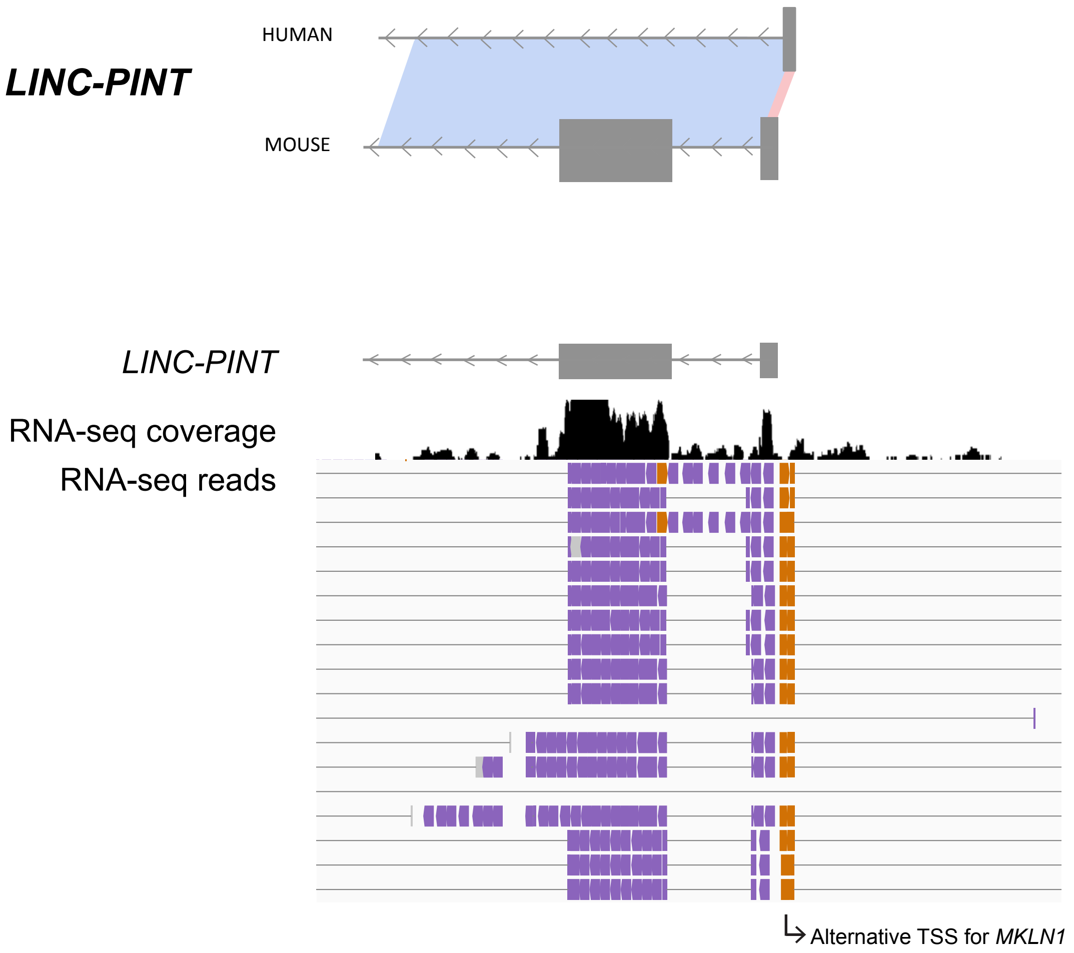

Figure S6. **Evolutionary alignment profiles are more robust than annotations for categorizing lncRNAs.** Top) Alignment profile of LINC-PINT, showing transcriptional homology only between the 5' exon of human and mouse. Bottom) IGV close-up of RNA-Seq alignments at the 5' end of LINC-PINT showing negative strand reads in purple and positive strand reads in orange. Positive strand reads represent an unannotated, alternative 5' end of MKLN1.

Supplementary Figure 7

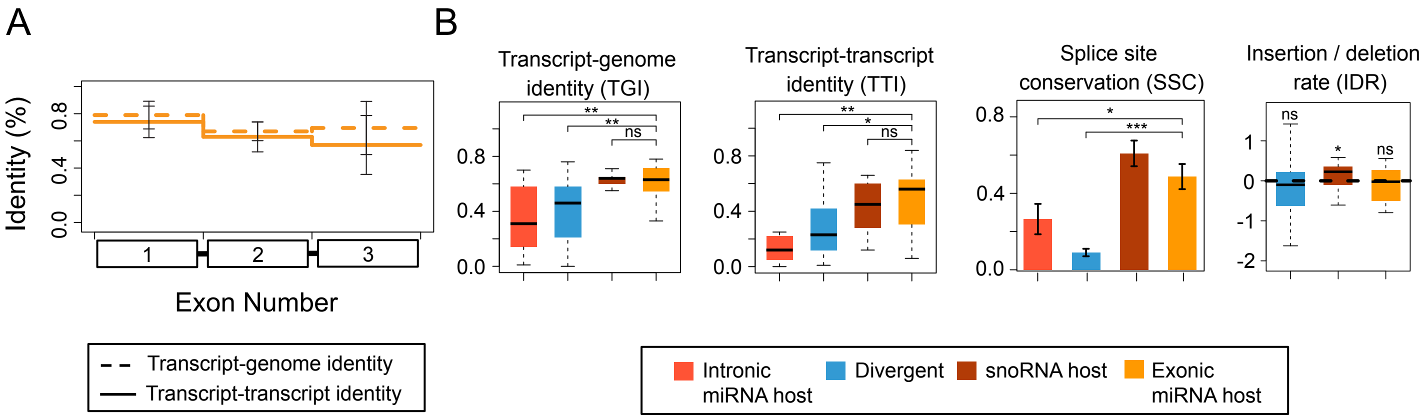

Figure S7. Exonic miRNA host genes are well conserved in sequence and transcriptional structure. A) Mean transcript-genome (TGI) (dotted lines) and transcript-transcript (TTI) (solid lines) identity of first three exons of host genes that harbor miRNAs in exons. B) Boxplots of TGI and TTI, barplot of splice site conservation, and boxplot of indel rate of intronic miRNA hosts (light orange), divergent (blue), snoRNA host (purple), and exonic miRNA hosts (dark orange). For all plots, two-sample t-test was used to test for significance, except one-sample t-test was used to test if mean of indel rate is deviated from 0. *** donates $P < 0.001$, ** denotes $P < 0.01$, and * denotes $P < 0.05$.

Supplementary Figure 8

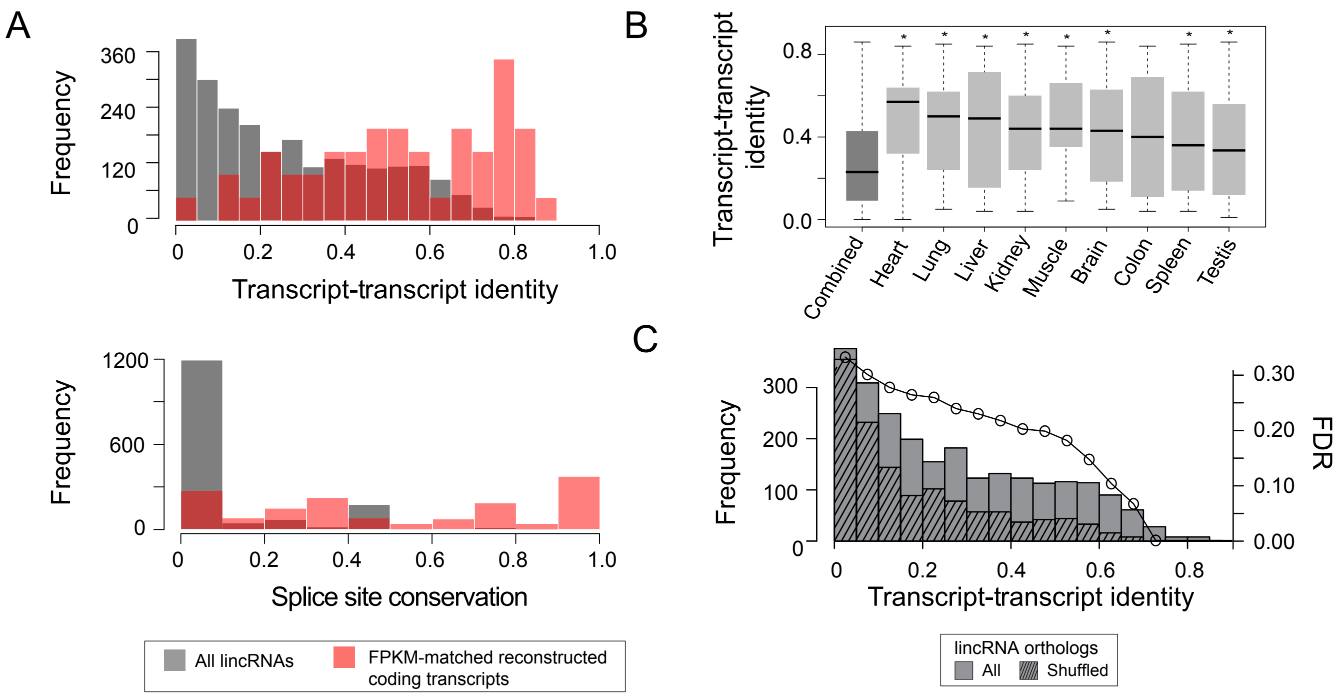

Figure S8. Poorly aligning lincRNA orthologs are likely artifactual results from large number of initial lincRNA transcripts. A) Histograms of transcript-transcript identity (TTI) (top) and splice site conservation (bottom) of all lincRNA orthologs (gray) compared to results from FPKM-matched set of reconstructed coding genes (red). B) Boxplots of TTI of all lincRNA orthologs compared to results when constraining initial set of lincRNAs to those expressed in matched tissues of human and mouse. * denotes $P < 0.05$ when compared to all lincRNAs (t-test). C) Histogram of TTI for all lincRNA orthologs (solid bars) compared to shuffled lincRNA orthologs (hashed bars) and estimated false discovery rate (y-axis).

Supplementary Table 2

Supplementary Table 2. Transcripts from combined lncRNA catalogs that likely harbor ORFs

Gene	Longest ORF (bp)	dN	dS	dN/dS	RNACode P-values
ENSMUSG00000053724	525	0.07	0.13	.541	4.177e-11
LINC00948 (MRLN)	141	0.04	0.21	.189	1.33e-08
LINC00890	273	0.01	0.09	.128	1.03e-08
LOC100507537	108	0.05	0.12	.456	1.71e-05
CDIPT-AS1	123	0.08	0.10	.451	4.799e-04
GQ868703	87	0.02	0.06	.266	5.00e-03
AK136239	60	0.03	0.08	.381	3.60e-02
AK094929	90	0.01	0.02	.273	3.60e-02

Table S2. Transcripts from combined lncRNA catalogs that likely harbor ORFs. The table lists transcripts in which slncky identified conserved ORFs that are also predicted to be coding by RNACode.

Supplementary Figure 9

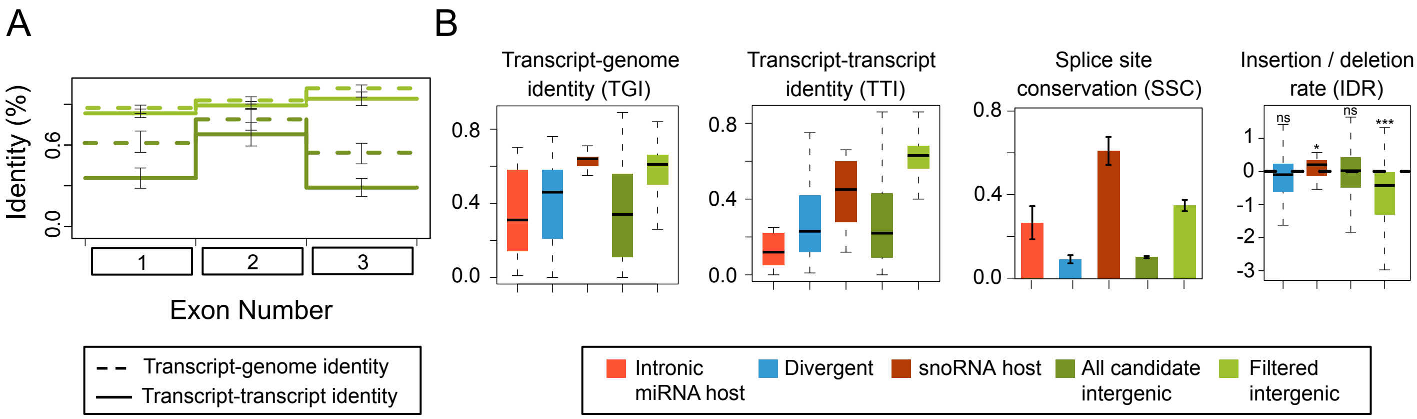

Figure S9. Conservation metrics of candidate and filtered intergenic lncRNA orthologs. A) Mean transcript-genome (TGI) (dotted lines) and transcript-transcript (TTI) (solid lines) identity of first three exons of candidate (dark green) and filtered (light green) intergenic orthologs. B) Boxplots of TGI and TTI, barplot of splice site conservation, and boxplot of indel rate of intronic miRNA hosts (light orange), divergent (blue), snoRNA host (purple), and candidate (dark green) and filtered (light green) intergenic orthologs. Because filtered intergenic orthologs were defined by higher TTI and SSC, we did not test for significantly higher TTI or SSC for this set. Instead we only indicate whether the mean of indel rate significantly deviated from 0 (t-test). *** donates $P < 0.001$, ** denotes $P < 0.01$, and * denotes $P < 0.05$.

Supplementary Table 3

Supplementary Table 3. Enrichment and depletion of repeat elements in lncRNA promoters

	Pluripotent lncRNA promoters		Necsulea, et al. lncRNA promoters						
	ES, mouse specific (n=291)	ES, mammalian conserved (n=48)	ES (n=829)	Brain (n=566)	Heart (n=352)	Kidney (n=828)	Liver (n=254)	Ovary (n=1170)	Testis (n=3379)
L1	9.19E-06	5.05E-02	8.22E-11	8.89E-07	2.85E-04	3.71E-07	7.27E-05	2.02E-16	2.90E-42
Low complexity	3.08E-01	5.75E-01	1.90E-05	6.00E-03	1.76E-03	1.71E-04	1.01E-01	2.76E-07	1.13E-06
Simple repeat	1.00E+00	4.33E-01	1.07E-01	1.79E-02	6.11E-02	7.58E-01	9.16E-01	2.88E-05	4.02E-07
Alu	3.14E-01	1.00E+00	4.18E-02	4.80E-01	1.26E-01	7.97E-03	5.68E-01	1.57E-02	2.62E-03
MaLR	1.00E+00	5.90E-02	1.76E-02	6.67E-01	1.45E-01	6.94E-01	7.95E-01	6.88E-01	1.46E-10
ERVK	1.65E-03	1.00E+00	4.10E-03	5.30E-02	7.86E-01	1.79E-02	8.66E-01	7.21E-02	1.71E-04
B4	1.31E-01	1.00E+00	7.14E-01	1.00E+00	7.19E-01	1.44E-02	5.95E-01	1.42E-01	2.96E-01
B2	3.76E-02	1.00E+00	3.09E-01	6.80E-02	7.86E-01	1.00E+00	4.92E-01	8.36E-01	4.11E-01
ERV1	2.97E-01	1.00E+00	3.24E-01	7.24E-01	4.85E-02	1.40E-01	3.82E-01	4.64E-01	6.97E-02

Table S3. Enrichment and depletion of repeat elements in lncRNA promoters.
The table shows Fisher's exact test P-values from comparing proportion of each repeat element present in lncRNA promoters to the proportion observed in GC-matched, random intergenic regions. Red denotes enrichment and blue denotes depletion
